# Supplementary figures and images for: Effects of six pyrimidine analogs on the growth of Tetrahymena thermophila and their implications in pyrimidine metabolism
Source: PLoS One. 2023 Sep 14;18(9):e0284309. doi: 10.1371/journal.pone.0284309 (PMC10501602; doi:10.1371/journal.pone.0284309)

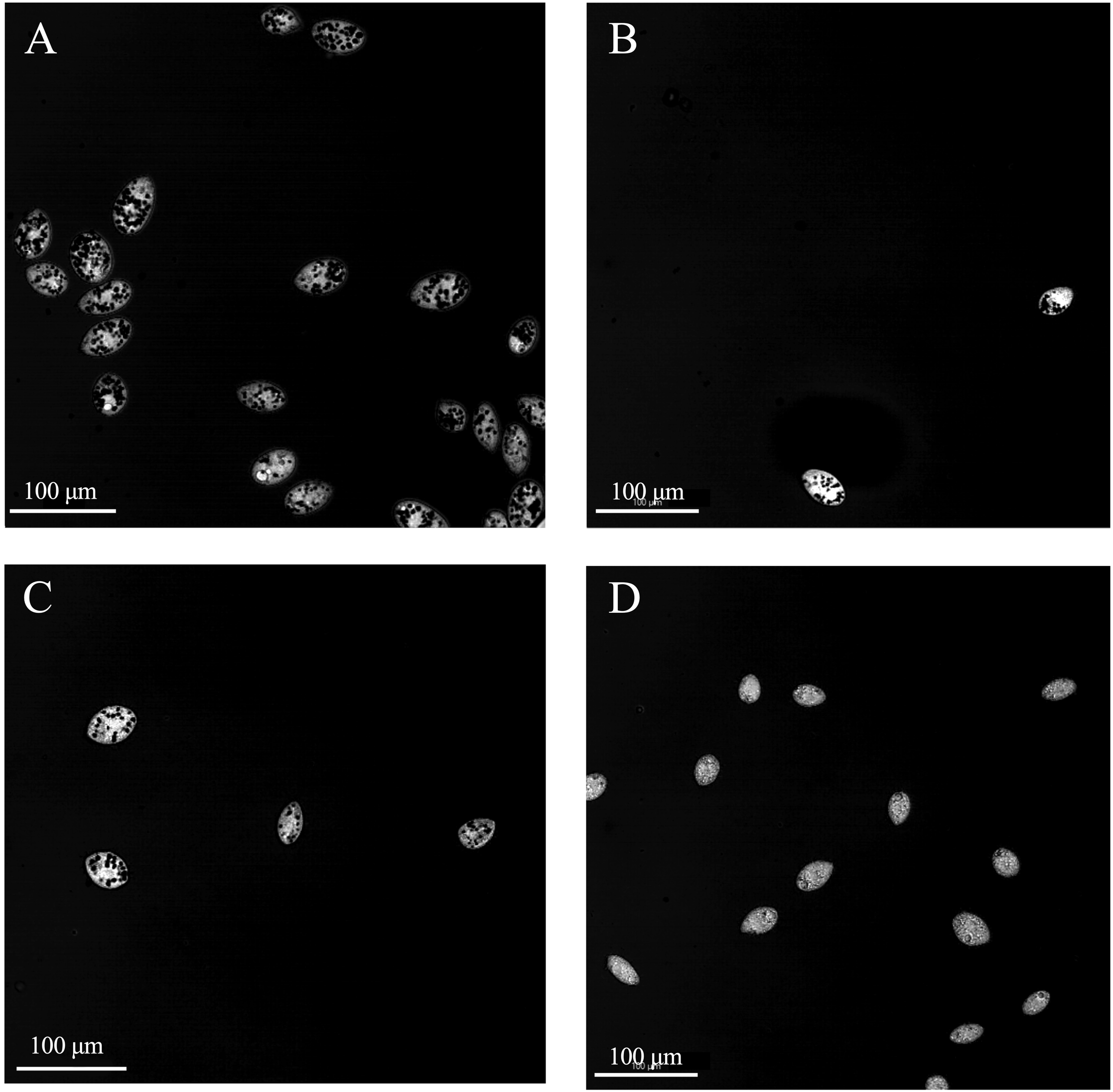

Supplement: S1 Fig — Micrographs taken at 20X magnification, 1.5X zoom. (A) CU428 at RT. (B) NP1 at RT. (C) 428 at 37°C. (D) NP1 at 37°C. The absence of dark vacuoles in NP1 at 37°C indicates a nonfunctional oral apparatus. (TIF) [file pone.0284309.s001.tif]

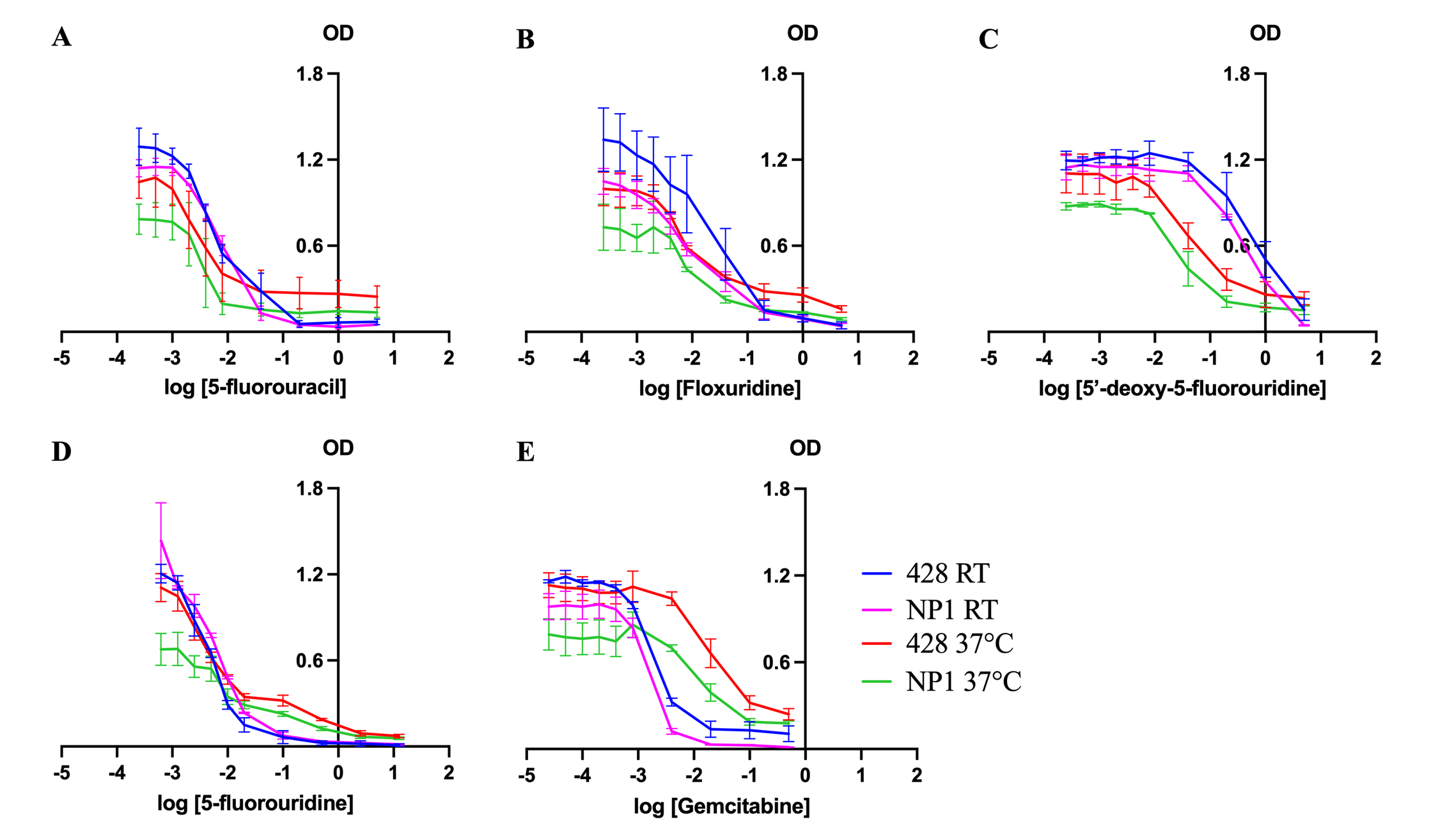

Supplement: S2 Fig — Data plotted ± SEM. (TIF) [file pone.0284309.s002.tif]

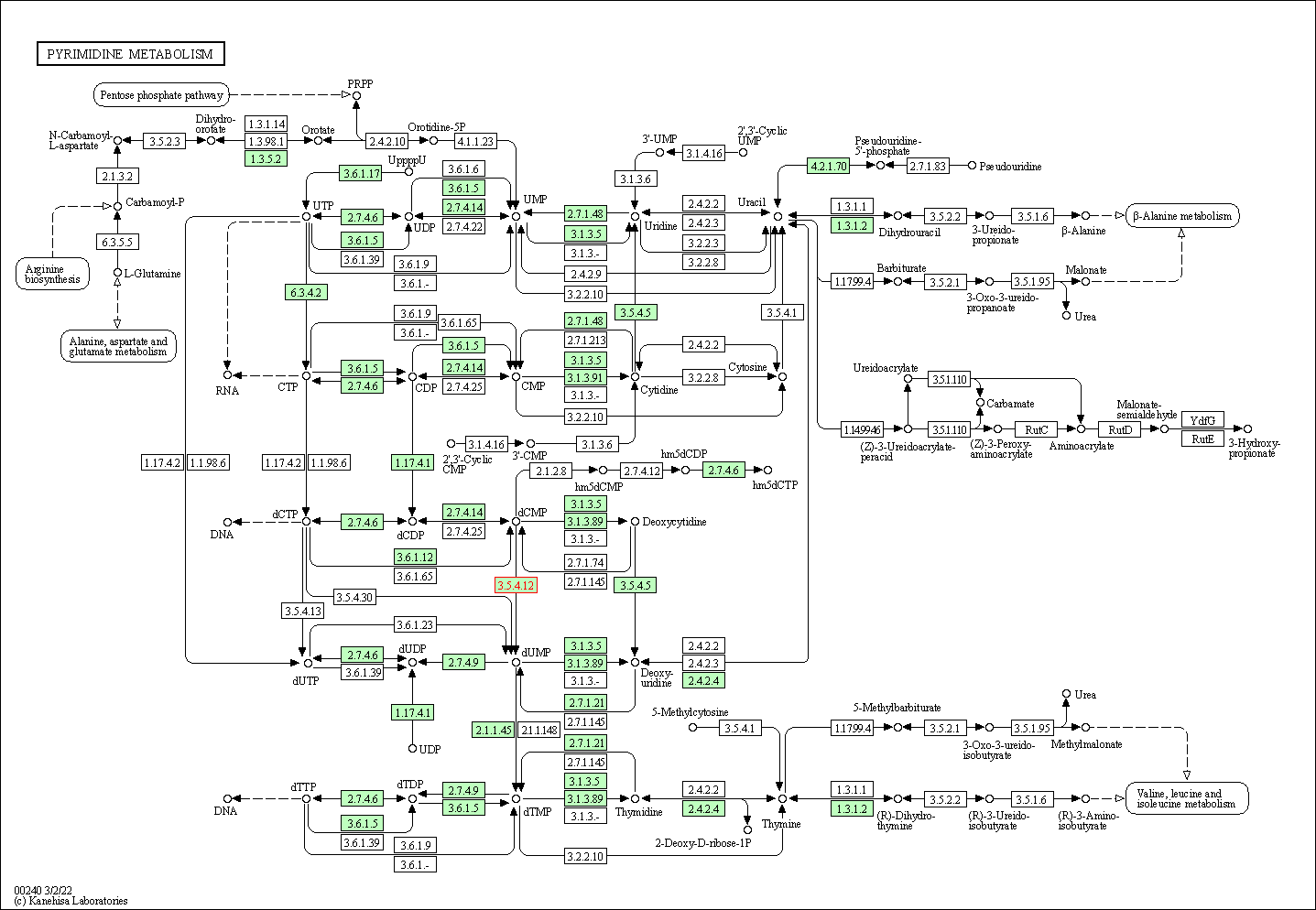

Supplement: S3 Fig — Homologous genes detected in the T. thermophila genome are highlighted in green. This diagram was created and downloaded from the KEGG database [19] by selecting the pathway type for T. thermophila. (TIF) [file pone.0284309.s003.tif]
