# Supplementary material for: Effects of six pyrimidine analogs on the growth of Tetrahymena thermophila and their implications in pyrimidine metabolism
Source: PLoS One. 2023 Sep 14;18(9):e0284309. doi: 10.1371/journal.pone.0284309 (PMC10501602; doi:10.1371/journal.pone.0284309)
Supplement: S1 Table — (DOCX) [file pone.0284309.s004.docx]

| **IC50 Value Comparison (CU428 v. NP1)** | ***P-*Value** |
| --- | --- |
| **Room Temperature** |  |
| 5-fluorouracil | .378 |
| Gemcitabine | .577 |
| Floxuridine | .307 |
| 5’-deoxy-5-fluorouridine | .548 |
| Fluorouridine | .884 |
|  |  |
| **37°C** |  |
| 5-fluorouracil | .787 |
| Gemcitabine | .213 |
| Floxuridine | .522 |
| 5’-deoxy-5-fluorouridine | .573 |
| Fluorouridine | .273 |
