# Supplementary material for: Effects of six pyrimidine analogs on the growth of Tetrahymena thermophila and their implications in pyrimidine metabolism
Source: PLoS One. 2023 Sep 14;18(9):e0284309. doi: 10.1371/journal.pone.0284309 (PMC10501602; doi:10.1371/journal.pone.0284309)
Supplement: S2 File — (DOCX) [file pone.0284309.s006.docx]

**Supplementary Materials and Methods**

Stock solutions of uracil, uridine, thymine, thymidine, and 5-methyluridine were prepared at a 20 mM concentration. The concentration of stock solutions of 5-fluorouracil, floxuridine, and 5’-deoxy-5-fluorouridine was 10 mM, whereas that of gemcitabine was 1 mM and 25 mM for 5-fluorouridine. All stock solutions were prepared in modified Neff’s medium.
